# Three-Dimensionally Printed Splints in Dentistry: A Comprehensive Review

**DOI:** 10.3390/dj13070312

**Published:** 2025-07-10

**Authors:** Luka Šimunović, Samir Čimić, Senka Meštrović

**Affiliations:** 1 Department of Orthodontics, School of Dental Medicine, University of Zagreb, 10000 Zagreb, Croatia; lsimunovic@sfzg.unizg.hr (L.Š.); mestrovic@sfzg.unizg.hr (S.M.); 2 Department of Prosthodontics, School of Dental Medicine, University of Zagreb, 10000 Zagreb, Croatia

**Keywords:** additive manufacturing, occlusal devices, digital workflow, SLA, DLP, orthodontics, temporomandibular disorder, biocompatibility, resin materials

## Abstract

Three-dimensional (3D) printing has emerged as a transformative technology in dental splint fabrication, offering significant advancements in customization, production speed, material efficiency, and patient comfort. This comprehensive review synthesizes the current literature on the clinical use, benefits, limitations, and future directions of 3D-printed dental splints across various disciplines, including prosthodontics, orthodontics, oral surgery, and restorative dentistry. Key 3D printing technologies such as stereolithography (SLA), digital light processing (DLP), and material jetting are discussed, along with the properties of contemporary photopolymer resins used in splint fabrication. Evidence indicates that while 3D-printed splints generally meet ISO standards for flexural strength and wear resistance, their mechanical properties are often 15–30% lower than those of heat-cured PMMA in head-to-head tests (flexural strength range 50–100 MPa vs. PMMA 100–130 MPa), and study-to-study variability is high. Some reports even show significantly reduced hardness and fatigue resistance in certain resins, underscoring material-specific heterogeneity. Clinical applications reviewed include occlusal stabilization for bruxism and temporomandibular disorders, surgical wafers for orthognathic procedures, orthodontic retainers, and endodontic guides. While current limitations include material aging, post-processing complexity, and variability in long-term outcomes, ongoing innovations—such as flexible resins, multi-material printing, and AI-driven design—hold promise for broader adoption. The review concludes with evidence-based clinical recommendations and identifies critical research gaps, particularly regarding long-term durability, pediatric applications, and quality control standards. This review supports the growing role of 3D printing as an efficient and versatile tool for delivering high-quality splint therapy in modern dental practice.

## 1. Introduction

Dental splints are removable intraoral appliances used across multiple dental disciplines to stabilize or protect teeth and jaws. In prosthodontics, occlusal splints are first-line therapy for bruxism and temporomandibular disorders (TMDs) [1,2,3]. The global prevalence of TMD is estimated at 34%, while bruxism (both sleep and awake) affects approximately 22.22% of the population. The global co-occurrence rate of bruxism and TMD is reported to be 17% [4]. In surgery and orthodontics, interocclusal splints help transfer orthognathic plans and maintain occlusion [5]. In restorative dentistry, printed guides assist endodontic access and tooth stabilization [6,7]. Traditional fabrication involves analog impressions and polymethyl methacrylate (PMMA) resin [8]. These time-tested but labor-intensive methods often rely on technician skill and are prone to error [9]. Limitations like shrinkage and residual monomer [8] have led to digital alternatives.

Digital workflows for splint fabrication now rely on computer-aided design (CAD) and computer-aided manufacturing (CAM) and 3D printing. Intraoral scanners and CAD software allow virtual splint design, replacing traditional impressions. Fabrication is performed via milling or 3D printing from biocompatible resins. Fully digital workflows can streamline laboratory steps and, in ideal settings, enable same-day splint delivery [10,11]. However, clinical reports indicate intraoral acquisition or print failures in 12–15% of cases—necessitating rescans or reprints that often add 30–90 min of chairside time and, in some instances, delay final delivery by a day or more [12]. Three-dimensional printing allows rapid, on-demand splint fabrication with minimal manual input, supporting complex geometries while consuming less material than milling [13]. Its adoption is rapidly growing across occlusal splints, aligners, and surgical guides due to its efficiency and precision.

A growing body of research supports the clinical viability of 3D-printed splints. In vitro studies show that their mechanical strength and wear resistance are somewhat lower than conventional acrylics but still meet ISO standards [14,15]. Systematic reviews from 2023 and 2024 confirm that 3D-printed splints demonstrate mechanically and chemically acceptable properties, achieving mean biaxial flexural strengths of 80 ± 11 MPa (range 60–95 MPa) and residual monomer releases of 0.03–0.08 µg/cm^2^—well within ISO 10993-5 cytotoxicity limits (cell viability > 90%) [14,15,16]. Clinical evaluations report patient outcomes and comfort on par with traditional splints [10], while printed surgical wafers in orthognathic procedures maintain clinically acceptable accuracy [5]. Ongoing prospective trials are further assessing long-term outcomes such as durability and patient satisfaction [16], reinforcing confidence in additive manufacturing for splint production.

Given the rapid development of digital fabrication, this review synthesizes current evidence on 3D-printed splints across prosthodontic, orthodontic, surgical, and restorative applications. We examine technologies, materials, and workflows, evaluating their accuracy and clinical effectiveness relative to traditional methods. By integrating data from laboratory and clinical studies, we identify key advantages, limitations, and knowledge gaps to guide both clinical implementation and future research. This review aims to synthesize current evidence regarding the fabrication, performance, and clinical utility of 3D-printed splints across dental disciplines, highlighting material innovations, limitations, and future directions.

**Methods of Article Selection:** We performed structured searches of PubMed, Scopus, and Web of Science for English-language articles published from January 2010 through April 2025 using the terms “3D printing,” “dental splint,” “occlusal guard,” “surgical guide,” and “orthodontic appliance.” We prioritized peer-reviewed original research (in vitro studies, clinical trials) and key technical reports and supplemented with narrative reviews for historical context. We excluded non–peer-reviewed conference abstracts, case reports, and articles without primary data. Reference lists of retrieved papers were hand-searched to capture any additional relevant studies.

## 2. Types of Dental Splints and Their Clinical Roles

### 2.1. Occlusal Splints (Stabilization Splints)

Occlusal splints (stabilization/night guards) are commonly used to treat bruxism and temporomandibular disorders (TMD), offering a conservative, reversible approach to reduce pain, protect the TMJ, and prevent tooth wear [3,17]. In bruxism, splints primarily protect the dentition from excessive wear, whereas in TMD, they often serve to deprogram masticatory muscles and unload the joint. Often a first-line therapy, they help to stabilize the bite and improve jaw-muscle function in affected patients [3,17]. Digital workflows allow for the scanning, design, and printing of splints within hours, eliminating traditional model work [18]. Early examples, such as Salmi et al. (2013), showed that printed splints provided good fit and patient satisfaction [12]. However, not all cases are suitable for 3D-printed devices—for instance, patients with severe bruxism may require more durable materials, such as milled PMMA, to withstand extreme parafunctional forces. More recent clinical trials report that printed stabilization splints match conventional ones in effectiveness, with improved comfort [12]. Similarly, short-term studies in sleep bruxism found that 3D-printed splints reduced muscle activity as effectively as acrylic versions [19]. Patient adaptation also varies: tolerance depends not only on fit but also on splint volume and whether an upper or lower appliance is used, so individual comfort assessments remain important. These findings support the clinical utility of 3D-printed splints in managing bruxism and TMD.

Because the STL file can be reprinted on demand, design iterations or remakes require only minutes of machine time.

Initial concerns regarding 3D-printed splints focused on their mechanical strength and resistance to wear. Compared to heat-cured PMMA, printed resins are generally more flexible and show slightly lower hardness and flexural strength [20,21]. Milled PMMA remains superior in toughness, while printed materials may absorb more water and show less complete polymerization [22,23]. Still, newer reinforced and flexible resins are narrowing this gap. Some reach strength values comparable to acrylics [1], and their flexibility may cushion impact forces [24]. Simulated wear and fatigue studies have confirmed that 3D-printed splints exhibit acceptable long-term durability under masticatory forces [25,26].

Biocompatibility studies show that well-cured 3D-printed splints release minimal residual monomer and are non-cytotoxic [26]. Wulff et al. (2022) found them comparable to PMMA in fibroblast tests [27], and Guerrero-Gironés et al. (2022) reported similar safety profiles between printed and conventional splints [26]. Overall, printed splints provide reliable clinical performance, with similar comfort and therapeutic outcomes as traditional versions [9,19]. While high-strength or hybrid materials may be better suited for severe bruxism, printed devices are an efficient and effective option for most patients.

### 2.2. Orthodontic Splints and Appliances

In orthodontics, the term “splint” can encompass a variety of devices used to guide or retain tooth positions. The traditional approach to fabricating such appliances involves either manual methods (e.g., vacuum-forming clear retainers over stone models) or the use of prefabricated components (e.g., standard brackets, wires, and acrylic bite plates). Three-dimensional printing is increasingly applied in this domain to improve customization and efficiency. However, most current clinical aligner systems still primarily use 3D printing to manufacture intermediate models rather than the aligners themselves. A prime example is the clear aligner therapy process—while the aligners themselves are usually thermoformed, the intermediate models for each tooth movement stage are virtually designed and 3D printed, enabling precise fabrication of the aligners. Recent advances have even explored direct 3D printing of aligners and orthodontic splints using biocompatible clear resins [28]. Xu et al. (2021) investigated a printable resin for orthodontic splints (retainers), finding that proper post-print cleaning and curing were critical to achieve optimal mechanical strength and minimize any cytotoxic residues, and noting that—since regulatory approval pathways differ by region—all resins intended for prolonged mucosal contact must undergo rigorous biocompatibility testing to ensure local safety standards are met [28]. Such research suggests that fully digital production of clear retainers—without the need for molds—is feasible, which could streamline orthodontic retention in the near future.

Beyond aligners, 3D printing has been applied to create custom orthodontic brackets and appliances. For instance, Krey et al. (2016) demonstrated a proof-of-concept for 3D-printed orthodontic brackets, showing that a personalized bracket system could be designed on a computer and fabricated additively [29]. However, technical challenges remain in achieving sufficient durability and deformation resistance for directly printed active elements—such as brackets or integrated elastic clips—that must withstand prolonged orthodontic forces and wear. The digital design allows fine-tuning of bite-opening height or tooth coverage in ways that are difficult with traditional fabrication. For example, a digitally designed bite splint can incorporate exact tooth channels and integrated springs or elastic hookups, which a printer can fabricate as one piece.

Three-dimensionally printed guides (splints) are increasingly used to accurately place temporary anchorage devices (TADs). Traditionally placed freehand, TADs risk inaccuracy; a printed splint fits over the teeth and guides the drill to the planned position. Mang de la Rosa et al. (2023) showed CAD/CAM guides significantly improved implant placement accuracy [30]. These splints are especially beneficial when working near critical anatomy or when symmetrical placement is required.

Orthodontic splints can also support orthognathic surgery, particularly during presurgical decompensation. Digital workflows enable accurate, printed transitional appliances. Caminiti and Lou introduced “clear aligner orthognathic splints,” where the final aligner guided both tooth alignment and surgical occlusion [31]. This innovative approach repurposes aligners as surgical wafers, blending orthodontic and surgical applications and demonstrating the versatility of 3D-printed aligner fabrication technology.

Overall, 3D printing in orthodontics enhances the fabrication of custom appliances, improving fit and patient comfort. Complex integrated appliances that once required laborious lab work (or were simply not possible) can now be built layer-by-layer. Patients benefit from appliances that are more precisely tailored to their anatomy—whether it is a nearly invisible clear retainer or a guide that makes a miniscrew insertion safer. As materials improve, we expect to see fully 3D-printed aligners and more orthodontic hardware (brackets, expanders, etc.) produced via additive manufacturing, further personalizing orthodontic care.

### 2.3. Surgical Splints (Orthognathic and Surgical Guides)

Surgical splints guide jaw positioning during orthognathic surgery. Traditionally made by articulating plaster models and manually forming acrylic wafers, this method is labor-intensive and error-prone. Digital workflows now allow virtual surgery and precise 3D-printed splints, which have become standard in bimaxillary cases. Shaheen et al. (2017) showed excellent agreement between digital plans and postoperative outcomes using printed final occlusal splints [5]. Similarly, Sarkarat et al. (2023) confirmed that 3D-printed maxillary repositioning guides were accurate enough for routine surgical use [32].

Digital workflows enhance surgical splint design by allowing virtual planning and in-software customization. Printed in resin, splints can be fine-tuned with features like model offset and occlusal coverage. Wang et al. (2023) found that a 100–200 µm offset maximized fit accuracy [33]. Other studies highlight that occlusal depth improves stability, but excessive coverage may hinder seating [34]. However, even with increased thickness, many resins remain prone to deformation under prolonged surgical conditions—especially high humidity or pressure—so material selection must be tailored to each procedure. These refinements improve intraoperative performance and are difficult to achieve with traditional methods.

The accuracy of these digital plans relies heavily on high-quality CBCT scans, precise segmentation, and correct occlusal registration; errors at any of these stages can compromise surgical outcomes. Mascarenhas and Makhoul (2021) showed that maxillary splints could be efficiently 3D-printed in-office for single-jaw surgeries, enabling rapid turnaround and last-minute adjustments [35]. These splints guided bone movement accurately and eliminated lab outsourcing delays. Digital plans can also be archived and reprinted if needed, offering a practical solution for smaller clinics.

3D printing allows novel surgical splint designs previously unachievable by hand. Kang et al. (2014) introduced a “Y-splint” with maxillary extensions for enhanced 3D stabilization [36]. Similarly, the clear aligner splint technique blends orthodontic and surgical workflows to improve comfort and alignment [31]. These examples highlight the creative potential of additive manufacturing in surgical applications.

Beyond orthognathic wafers, custom splints are used in other surgical contexts. In trauma and reconstructive cases, 3D-printed splints help maintain pre-injury occlusion or reposition segments. Studies show their success in guiding complex fracture healing or infant cleft molding [37,38,39].

Another surgical use is in guided implantology—while typically called surgical guides rather than splints, these devices share similarities. They fit over remaining teeth or alveolar bone and guide implant drills to the planned positions. Three-dimensional printing has made such guides routine in implant surgery. Although implant guides are usually discussed separately from splints, they reinforce the point that additive manufacturing can produce highly accurate intraoral guides for a variety of surgical procedures, improving precision and outcomes [40].

An elegant clinical illustration is the Hungarian case series by Czako et al., in which patient-specific 3D-printed guides enabled safe styloid-process resection for Eagle’s syndrome; mean angular deviation of resections was <2°, and no neurovascular injuries were reported [41]. Their work broadens the clinical canvas from removable splints to hard-tissue navigation and underscores the importance of image-based CAD in addressing orofacial pain with anatomical roots beyond occlusion alone.

3D printing has transformed surgical splints by enabling accurate, customizable designs that reliably transfer planned jaw movements [33]. Rapid in-house production, improved fit, and creative shapes (e.g., cut guides or reinforcements) are key benefits. Although resin splints may be less robust than acrylics, design strategies such as added thickness mitigate distortion risks in complex or prolonged surgeries. These advances have made 3D-printed splints standard in orthognathic surgery and increasingly common in trauma and pediatric applications.

### 2.4. Restorative Splints and Guides

In restorative dentistry, the term “splint” is used in various contexts. It can refer to periodontal splints, which stabilize loosened teeth (for example, bonding teeth together to distribute forces), or to guide stents that assist in executing restorative procedures. 3D-printed guides in restorative and endodontic procedures serve as splints that direct precise modifications. Examples include guides for tooth prep, post removal, or calcified canal access [7,42,43,44].

In full-mouth rehabilitation, printed diagnostic splints help test new vertical dimensions and tooth shapes before final restorations [45]. These overlays simulate outcomes, guide patient feedback, and can be revised digitally. Once optimized, the final prosthetic work follows. This approach increases precision and predictability in complex prosthodontic cases [46].

Restorative splints guide or stabilize structures during procedures such as endodontics, crown prep, or soft tissue management. Using 3D scans, clinicians design guides to translate treatment plans with precision. Reports confirm success in enhancing accuracy and preserving tooth structure [7,42]. As CAD/CAM becomes standard, these splints offer “roadmaps” for executing complex procedures and improving outcomes. Rather than passive stabilizers, printed splints now actively support clinical treatment execution in restorative dentistry.

## 3. Three-Dimensional Printing Technologies and Materials Used in Splint Fabrication

### 3.1. Overview of 3D Printing Technologies in Dentistry

Several 3D printing technologies are used in dentistry. SLA and DLP, the most common for splints, use light to polymerize liquid resin layer-by-layer and produce high-resolution, accurate appliances [47]. However, not all SLA/DLP resins are inherently biocompatible—proper certification and thorough post-processing (cleaning and curing) are essential to remove residual monomers that may irritate mucosa. SLA employs a UV laser, while DLP projects an entire layer at once. Both are favored for their precision and compatibility with biocompatible resins. FDM, which extrudes thermoplastic filaments, is less suitable due to rough surface finishes and lower resolution, making it better for models than intraoral splints [48]. Nevertheless, FDM remains effective for diagnostic models, preliminary surgical guides, or educational anatomical replicas where ultra-fine surface detail is not critical. Material jetting (e.g., PolyJet) allows multijet resin deposition with fine detail, useful for complex designs. For instance, a triple-jetting system was used to fabricate orthodontic splints with properties comparable to PMMA [24]. These digital methods allow fast, reproducible splint production directly from CAD, streamlining the clinical workflow from scan to appliance.

3D printing offers efficiency and improved clinical outcomes. CAD enables control over splint thickness and occlusal geometry, enhancing fit and function [49]. Because splints are printed from digital models, identical copies can be easily produced. Clinical trials report greater patient comfort with 3D-printed splints—one study showed a mean comfort score of 15 vs. 42 for conventional appliances [50]. The smoother fit and reduced bulk likely improve compliance. Additionally, once a printer is calibrated, splints can be fabricated rapidly with minimal labor—an advantage in urgent cases like surgical wafers or lost night guards.

Despite its advantages, 3D printing remains a relatively new and evolving technology in dental practice [1]. There is a learning curve for clinicians and technicians to master CAD and printer operation. Additionally, the initial investment in a dental 3D printer and materials can be significant, which has been cited as a barrier to widespread adoption in some practices (the cost of the printer and resin, plus the need for post-processing equipment like curing units) [1]. In summary, SLA and DLP printing have emerged as leading technologies for dental splint fabrication, offering high precision and customizability. Other methods, such as FDM, play a minor role due to accuracy limitations, while material-jetting systems provide a niche high-end solution. With increasing clinician experience and improvements in printer affordability, these technologies are becoming integral to modern dental workflows for splint therapy.

### 3.2. Material Classes for 3D-Printed Splints

Most printable splint resins are methacrylate-based photopolymers that polymerize into rigid acrylic-like structures. Common chemistries include urethane dimethacrylate (UDMA) and other acrylic monomers with photoinitiators. These aim to mimic the stiffness and strength of heat-processed PMMA [13]. Flexural strength typically ranges from ~50 to 100 MPa, approaching—but often not equaling—that of PMMA. One newer formulation achieved comparable strength and hardness [1]. Although some resins show higher water sorption or slightly incomplete polymerization, improvements in chemistry are closing the performance gap. Overall, current printed materials are clinically adequate but still evolving toward the robustness of long-standing analog resins.

New “flexible” or thermo-resilient resins expand splint options. These soft materials, such as those that soften at mouth temperature, offer improved comfort while remaining functional. In one trial, a thermo-flexible printed splint showed equivalent clinical performance to a hard milled one after three months of use, with no fractures [50]. Patients reported good tolerance. Another FDA-cleared material, KeySplint Soft, yields pliable but tough appliances. These flexible splints exhibit higher ductility and fracture toughness than rigid ones, with only slight strength trade-offs [51]. Such materials are ideal for interim use or for patients sensitive to rigid appliances.

Emerging 4D shape-memory photopolymers add an even smarter tier of functionality. These resins can be programmed to deform under intraoral temperature or pH, then return to their original geometry—opening the door to self-adjusting occlusal contacts and stimulus-responsive scaffoldings. Thurzo and Varga (2025) recently demonstrated AI-guided personalization of such shape-memory methacrylate networks for oral soft-tissue scaffolds, highlighting the near-term translational potential of “4D-printed” dental appliances [52]. Nevertheless, published data remain benchtop only; no clinical trials have yet verified long-term safety or mechanical stability, underscoring the need for controlled in vivo studies before routine use.

In addition to photopolymer resins, there are thermoplastic materials used in subtractive or other additive processes that warrant mention. Polycarbonate is one such material: it is a tough, transparent thermoplastic that has been used to mill splints, and experimental work has also produced splints by extruding or printing polycarbonate. Milled polycarbonate splints offer high impact resistance and flexural strength, but questions have been raised about chemical safety due to potential bisphenol-A (BPA) content [53,54]. A study comparing milled versus 3D-printed dental polycarbonate found that both released only trace amounts of BPA, though careful post-processing was required for the printed version [55]. This suggests polycarbonate could be a viable splint material in the future, offering durability beyond methacrylate resins, if provided biocompatibility is ensured. Other thermoplastics like polyethylene terephthalate glycol (PET-G) or ABS have occasionally been explored via FDM printing for making simple night guards or surgical splints, but these materials are not specifically approved for long-term intraoral use and tend to lack the precise fit achievable with resin printing.

Furthermore, there is active research into reinforcing and modifying resin compositions to enhance their mechanical and biological performance. For example, one approach is incorporating nanofillers into the resin matrix. Recent work investigated adding graphene nanoplatelets to a 3D-printable splint resin to improve its strength. The modified resin showed a significant increase in biaxial flexural strength, indicating that nanoparticle reinforcement can toughen the printed splint, although the effect on biocompatibility needed careful evaluation [56]. Another innovative modification is the incorporation of bioactive additives. For instance, researchers have added fluoride-releasing bioactive glass fillers to methacrylate resin in order to create splints that could potentially help remineralize enamel or reduce bacterial activity. These experimental materials have shown the capacity to release fluoride ions while maintaining acceptable strength, pointing toward future splints that are not just passive devices but could confer therapeutic benefits [57]. In summary, material options for 3D-printed splints range from rigid acrylic-like resins to flexible polymer formulations, and ongoing developments aim to improve their mechanical resilience, patient comfort, and even biological functionality.

### 3.3. Post-Processing Requirements for Printed Splints

After a splint is fabricated in a 3D printer, it must undergo critical post-processing steps to reach its final form and properties. Post-curing and cleaning are mandatory to ensure biocompatibility and mechanical strength. Most commonly, rinsing with IPA in two baths is sufficient, followed by UV light curing. Studies show overexposure can leach material, while proper curing improves flexural strength and reduces toxicity [28,55,58,59].

The next crucial step is post-curing polymerization. Printed splints are usually cured in a specialized light-curing unit, often with UV or broad-spectrum light, sometimes combined with heat. Proper post-curing significantly increases the degree of conversion of the resin—that is, it drives remaining monomers into polymer—thereby hardening the splint and improving its mechanical properties. Inadequate post-curing may result in reduced mechanical strength and increased residual monomer release. Research has demonstrated that post-curing under an inert gas atmosphere (such as nitrogen) can further enhance the polymerization because it prevents oxygen inhibition on the surface. In one experiment, identical splint specimens were post-cured in air versus in a nitrogen-purged chamber; the nitrogen-post-cured samples showed higher flexural strength and hardness due to more complete curing at the surface layer [51]. Inadequate post-curing not only leaves the splint softer but also with a higher residual monomer content, which can cause irritation or cytotoxicity in vivo. In practice, some modern curing units for dental resin printing now incorporate inert gas or vacuum settings to take advantage of this effect. The intensity and duration of the curing light are also important—manufacturers typically recommend a specific curing cycle (for example, 10–30 min of high-intensity 405 nm LED light). Following those instructions is necessary to achieve the material properties reported by the manufacturer. Finishing steps—including support removal, polishing, and, when necessary, sterilization—are outlined in Table 1. These steps are critical to ensure biocompatibility, durability, and patient comfort [28,55,58,60,61,62,63,64,65,66,67].

### 3.4. Limitations and Challenges of 3D-Printed Splints

While 3D printing technology for splints is highly promising, there are several limitations and challenges that practitioners and researchers have identified. In theory, different classes of resins exist: night guard resins, orthodontic retention resins, and surgical splint resins—each with varying mechanical and biological properties, although today many manufacturers use essentially the same base formulations across these categories. One major consideration is the mechanical performance of printed splints compared to those made by traditional methods. Numerous studies indicate that, although printable resins are improving, they often exhibit lower values in key properties such as hardness, stiffness, and wear resistance relative to conventional heat-cured or milled PMMA splints. A 2023 systematic review collating data on various splint materials found that the PMMA-based conventional splints still showed the highest flexural strength, modulus, hardness, and fracture toughness among all groups; by contrast, the additively manufactured (3D printed) splints generally had higher water sorption and a lower degree of cure (as indicated by a greater fraction of unreacted double bonds) [13]. This incomplete degree of conversion in printed resins can lead to a less rigid polymer matrix and the presence of more residual monomers. Proper post-processing—including optimized curing time, light intensity, and meticulous cleaning—is critical, as these factors significantly influence final properties like hardness, conversion rate, and cytotoxicity. Another study comparing pressed, milled, and 3D-printed resins for occlusal devices reported that the printed resin had about 20–30% lower flexural strength than the others, reflecting the same trend [68]. In clinical terms, this means printed splints might be more prone to cracking or deforming under heavy occlusal forces, especially in patients with severe bruxism. Moreover, individual functional assessment—considering occlusal force direction, contact points, and nocturnal parafunctional habits—is essential to predict longevity and performance. However, it should be noted that most printed splint materials do meet the minimum ISO standards for dental polymers and have been successfully used in practice. The somewhat reduced strength is usually not a problem for normal use, but it remains a point of active improvement for material scientists.

Related to strength is the wear resistance of printed splints. Some occlusal splints are subject to considerable wear over time due to parafunctional grinding forces. Conventional hard acrylic has a long track record of reasonable wear performance (gradual surface wear but usually no deep abrasion in the short term). The wear behavior of newer printed materials is still being characterized. A systematic review on wear of splint materials manufactured by various methods concluded that subtractive materials (like milled PMMA) and conventional cured resins tended to have better wear resistance than many early printed resins, which showed faster material loss in two-body wear tests [69]. Additionally, surface hardness correlates with wear—since printed splints often have lower hardness, they may wear down more quickly. There is ongoing research to quantify this: for example, Lawson et al. (2025) tested the wear resistance of several 3D-printed splint materials and found variability between brands, with some newer formulations approaching the wear resistance of milled PMMA, while others showed noticeable volumetric loss in wear simulations [70]. For now, clinicians should be aware that a 3D-printed night guard might need replacement sooner than a traditional one if the patient has heavy grinding habits, although exact longevity will depend on the specific resin and patient use patterns.

Another challenge lies in the accuracy and fit of printed splints. Achieving a perfect fit is crucial for splint efficacy and comfort. Several factors in the printing process can introduce slight inaccuracies: polymerization shrinkage of the resin, the layer-by-layer building (which can cause stair-step artifacts on inclined surfaces), and the need for support structures that may distort the appliance if not placed optimally [71]. Studies evaluating the dimensional accuracy of printed splints versus milled splints have shown mixed results but generally indicate they are on par with each other clinically, with some nuances. Reymus et al. compared the accuracy of milled vs. three-dimensionally printed bite splints and found that milled splints had slightly higher trueness (closer to the digital design), but printed splints had high precision, and any deviation was small and consistent [13]. Importantly, they concluded that both methods produced clinically acceptable results, with mean discrepancies in the range of tens of microns. The orientation of printing can significantly affect accuracy: printing a splint flat (horizontal) vs. upright (vertical) changes how the layers stack and how strain is released. In the above study, horizontal orientation yielded better overall trueness, while vertical orientation gave slightly better consistency in certain dimensions. Another investigation specifically focusing on build angle found that printing splints at a 120° angle improved certain fit metrics compared to 90° or 45°, highlighting that optimal orientation can minimize distortion [64]. Furthermore, software settings like model offset (a deliberate slight enlargement of the digital model to account for expected shrinkage) are important. Wang et al. demonstrated that using a proper offset value when designing the splint can greatly enhance the precision of the fit; too little offset and the splint may be tight, and too much and it may be loose [33]. For clinicians, this means following manufacturer recommendations on print orientation and any compensation factors, as well as iterative evaluation of fit, is necessary to consistently obtain well-fitting appliances.

Biocompatibility and chemical safety form another area of concern. All dental materials in the mouth must be non-toxic and non-irritating. Traditional heat-cured or auto-polymerizing acrylics can leach monomers like methyl methacrylate if not cured fully, and similarly, printed resins can leach photoinitiators or oligomers if the curing is incomplete [72]. In vitro cytotoxicity tests have raised caution that some 3D-printed splint materials, in their uncured or partially cured state, can reduce cell viability. For example, Wulff et al. tested eluates from several printed splint resins on fibroblast cell cultures and found measurable cytotoxic effects, especially from freshly printed samples, whereas well-post-cured samples showed greatly reduced cytotoxicity [27]. This reinforces the necessity of proper post-processing (as discussed in Section 3.3). Another related issue is the potential allergenicity of certain resin components; a small percentage of patients could exhibit sensitivity to methacrylates. Thus far, clinical reports of adverse reactions to printed splints are rare, but practitioners should be mindful of this possibility, especially in patients with known acrylic allergies. Water sorption of printed resins is typically higher than that of fully dense PMMA (they can absorb water and saliva over time), which might not only slightly alter their dimensions but could also facilitate slow release of any residual uncured compounds. Wedekind et al. analyzed the eluate from printed vs. milled vs. conventional splint materials and detected that the printed resin (SheraPrint-Ortho Plus) released small amounts of various co-monomers in solvent, whereas the milled and conventional materials released virtually none in water—although in water at 37 °C the printed splint primarily leached a tiny amount of one monomer, tetrahydrofurfuryl methacrylate (THFMA), below toxic levels [8]. Their work suggests that, in a worst-case scenario (solvent environment), printed splints could release more compounds than other types, but in actual intraoral conditions the levels are likely minimal and safe if the device is properly cured. Nevertheless, the long-term biocompatibility of 3D-printed splints is still being researched, and regulatory bodies have typically cleared these materials for intraoral use based on short-term testing. Long-term data (spanning several years of continuous use) is not yet abundant.

Finally, there are practical challenges to consider. One is the requirement for additional equipment and training, as mentioned earlier. Not every dental clinic or lab has the facilities to perform high-quality 3D printing and the subsequent curing and polishing; this can centralize production in larger labs or necessitate capital investments. Another challenge is consistency: 3D printers must be regularly calibrated and maintained. Factors like resin batch differences, printer light source intensity, and even ambient temperature can subtly affect outcomes. For critical applications like surgical splints, some clinicians still prefer the familiarity of conventional methods or milled splints unless the 3D printer’s accuracy has been well validated. There have been reports that certain 3D-printed surgical wafers for jaw surgery showed slight inaccuracies that needed intraoperative adjustment, underscoring the need for meticulous quality control in those high-stakes uses [32]. Another practical limitation is that the printed resin splints are typically one uniform material; unlike some traditional approaches where a soft liner can be added to a hard splint, multi-material printing (e.g., a soft inner layer with a hard outer shell) is not yet common with the current generation of dental printers (except on expensive PolyJet machines). This means a printed splint may not have the same level of shock absorption or dual-material advantage that a lab-fabricated dual-laminate night guard might offer.

Despite encouraging reports of clinical performance, our review revealed moderate to high heterogeneity in mechanical outcomes across 3D-printing studies. In published meta-analyses of flexural strength, between-study I^2^ consistently exceeded 60% for most resin classes, reflecting differences in polymer chemistry, printer type, post-curing regimes, and testing methods. A sensitivity analysis indicates that resins subjected to post-curing under inert nitrogen or extended UV exposure achieve flexural strengths within 10–15% of heat-cured PMMA, whereas “quick-cure” protocols without oxygen control often yield strengths 15–25% lower than PMMA benchmarks (e.g., flexural strength 75 ± 10 MPa vs. 110 ± 5 MPa for PMMA) [61,68]. Clinicians should therefore interpret manufacturer-reported values with caution, consult independent laboratory data when available, and specify resin type and post-processing parameters in both research reports and clinical records.

In conclusion, 3D-printed splints represent a significant advancement in dental appliance fabrication, but like any technology, they come with limitations that must be managed. Ongoing improvements in resin chemistry are addressing mechanical weaknesses (for instance, new resins with higher conversion and added tougheners are already narrowing the gap with PMMA). Improved printer hardware and software algorithms are enhancing accuracy and reproducibility (e.g., auto-compensation for shrinkage, optimized support strategies). For the clinician, understanding these challenges—from ensuring a proper cure to appreciating that a printed device might wear a bit faster—is key to successfully implementing 3D-printed splints in practice. With careful case selection and adherence to recommended workflows, the current generation of printed splints can perform equivalently to conventional devices for many applications while offering unique advantages in customization and efficiency. Researchers and manufacturers are actively addressing the remaining challenges, so it is likely that in the near future 3D-printed splints will further improve in strength, longevity, and overall reliability, solidifying their role in dentistry’s arsenal for managing TMDs, bruxism, and surgical orthodontics (Table 2).

## 4. Digital Workflow for Splint Fabrication

Modern splint fabrication has shifted from analog techniques (impressions, stone models, and hand-crafted acrylic) to a fully digital workflow. This workflow typically involves four phases—digital impression acquisition, computer-aided design (CAD), additive manufacturing (3D printing), and post-processing—each offering improvements in accuracy and efficiency over traditional methods. Studies consistently show that a well-implemented digital process can produce splints with equal or greater fit accuracy and patient satisfaction while significantly speeding up production and reducing material waste [23]. Below, we detail each phase of the digital workflow and compare it to conventional practices in terms of clinical efficacy and efficiency.

### 4.1. Scanning and Data Acquisition

Digital fabrication begins with capturing the patient’s dental anatomy and bite relationship using intraoral scanning. An intraoral scanner (IOS) creates a precise 3D model of the upper and lower arches, replacing the need for physical impressions. Recent improvements in IOS technology have made full-arch digital impressions comparable in accuracy to conventional impressions for splint fabrication [73]. In fact, a systematic review found that modern scanners can achieve full-arch trueness within ~100–200 μm of conventional methods, indicating that the long-standing limitations of digital impressions are largely overcome [74]. For clinicians, this means a well-calibrated IOS can capture the arches and occlusal relationships with sufficient fidelity for splint design.

Advantages over analog: Patients avoid traditional alginate or silicone impressions, which can induce gagging or distortion upon removal. Instead, the IOS painlessly records the teeth and bite in minutes, and the data is immediately available—eliminating the time needed to pour plaster models. In one educational study, fourth-year dental students with no prior digital experience averaged about 17 min to complete a full-arch IOS scan, demonstrating that even novice users can obtain accurate digital models in a reasonable time [18]. Experienced clinicians report much faster scan times, and, importantly, digital scans bypass potential errors from impression material shrinkage or plaster expansion. The resultant STL files precisely represent the dentition without the dimensional changes that can occur in physical model fabrication.

After scanning each arch, the bite or occlusal relationship is recorded. This can be performed by scanning the patient in maximum intercuspation (e.g., a buccal bite scan aligning upper and lower models) or by scanning an interocclusal record (centric relation) [75]. The digital workflow ensures that the maxillary and mandibular scans are correctly articulated in the virtual environment. In contrast, the conventional process requires a separate bite registration material and manual mounting of models on an articulator. Digital articulation is typically faster and less prone to mounting errors. Moreover, digital impressions are easier to store and transfer—the 3D files can be archived indefinitely or sent electronically to a lab/CAD technician, whereas physical casts require storage space and can be damaged over time.

It should be noted that full-arch intraoral scanning has a learning curve. Operator experience can impact the outcome: a proper scan path and technique are needed to capture all tooth surfaces without holes or stitching errors [73]. In a student cohort, those with prior CAD/CAM exposure performed scans more efficiently and produced models that needed fewer adjustments [18]. Nonetheless, even beginners found the digital workflow feasible, and 79% rated the overall process (scanning through delivery) as “very good” or “satisfactory” in a clinical exercise [18]. These findings suggest that digital data acquisition is clinically attainable and can yield results on par with traditional impressions once the user is adequately trained.

Beyond standard intraoral scanning, advanced data acquisition can integrate additional records for complex cases. For example, in patients requiring jaw repositioning (e.g., for temporomandibular disorder therapy), cone-beam CT scans or jaw motion tracking may be incorporated alongside the intraoral scans. Nota et al. [76] demonstrated a full digital workflow to establish a new condylar position by merging CBCT data with scanned models; specialized software automatically repositioned the mandible to an ideal therapeutic position before splint design [76]. This kind of data integration is difficult in an analog workflow, but digitally it allows clinicians to pre-visualize and plan splint therapy with unprecedented precision. In summary, digital data acquisition provides a highly accurate virtual model of the patient, forming the foundation for the subsequent CAD phase and offering improvements in convenience, accuracy, and diagnostic insight compared to traditional methods.

### 4.2. Design and CAD Software

Once the digital impressions are obtained, the next phase is computer-aided design of the splint. Using specialized dental CAD software, the technician or clinician virtually designs the occlusal splint on the 3D models. Common platforms for splint design include 3Shape Splint Studio, Exocad’s splint module, and standalone tools (e.g., Blenderfordental or D3Splint). These programs provide workflows to delineate the splint’s geometry, ensuring a proper fit and desired occlusal scheme, though movable soft tissues (e.g., palatal mucosa or vestibular areas) may not be fully captured by intraoral scans, requiring manual finishing or minor clinical adjustments.

The typical CAD steps are as follows:

- Model preparation: The scanned upper and lower arches are imported and aligned in the correct occlusal relationship. The software may allow verification of contact points in the starting occlusion. Any artifacts or distortions in the scan can be trimmed or corrected at this stage.
- Defining splint margins: The user outlines the intended coverage on the teeth and gums. For a full-coverage stabilization splint (Michigan-type), this usually means covering all teeth of one arch (commonly the upper) up to a determined border on the buccal/labial and lingual sides. Undercuts can be optionally blocked out in the software to adjust retention—some workflows block undercuts minimally to ease insertion, whereas others intentionally engage certain undercuts for a snug fit.
- Setting thickness and extensions: The software then generates an initial splint shell over the chosen teeth with a uniform thickness (often 2–3 mm, or as required for strength). The user can adjust parameters such as overall thickness, occlusal pad thickness, and whether to cover the incisal edges. Digital design ensures that a uniform minimum thickness is achieved, which is important for splint durability—this is harder to control precisely in a purely manual process. Moreover, CAD makes it easy to export multiple design variants (e.g., differing occlusal heights or reliefs for endodontically treated teeth) to test patient adaptation or plan gradual bite adjustments.
- Occlusal surface design: Using the opposing arch model, the CAD software will generate the occluding surface of the splint. Most splint software incorporates a virtual articulator or dynamic occlusion tool to achieve the desired contact pattern. For example, 3Shape’s Splint Studio can automatically create a flat occlusal plane or a cuspid-guided surface and show the distribution of contacts [67]. The designer can visualize where the opposing teeth would contact the splint and adjust these areas. The goal for a stabilization splint is usually even, simultaneous contact of all opposing posterior teeth on the splint in centric closure, with slight canine-guided disclusion in excursions [17]. In CAD, achieving this can be performed by removing high spots (visible as red pressure areas in the software) and refining the surface until desired contacts are observed (often verified by the software’s color mapping of contact strength). This digital occlusal adjustment is analogous to the clinical remounting and grinding of spots on an acrylic splint, but it is performed virtually. Digital analysis tools like the T-Scan Novus system can even be used after fabrication to fine-tune contacts, as shown in one case report where the splint’s occlusion was adjusted guided by digital bite force data [77]. In practice, a well-balanced design in CAD should reduce the amount of chairside adjustment needed when the splint is delivered.
- Exporting the design: After finalizing the shape, the splint design is exported (typically as an STL file) for manufacturing. At this point, the digital design can be virtually assessed for any undercuts or tight areas (software often provides a cross-sectional view to check the fit on teeth). If the design is performed in-office, the dentist can 3D print it immediately; if performed by a lab, the file is sent back for fabrication. Notably, the digital design can be saved indefinitely, allowing easy reproduction of the splint if it is lost or broken, or quick modifications to create a new iteration. This is a major advantage over traditional methods, which would require starting from scratch or physically storing models that might be discarded or damaged.

Comparisons and advantages: CAD splint design offers a level of precision and repeatability that surpasses manual techniques. In the analog process, a technician would typically wax up an appliance or use autopolymerizing acrylic on a model, then adjust it on an articulator—a process highly dependent on individual skill. By contrast, digital design uses accurate computational tools to ensure even spacing, uniform material thickness, and proper occlusal contacts. The entire shape is visualized in software, reducing uncertainty. As one example of the capability of CAD, Liu et al. (2025) [78] reported a novel digital design of an anterior repositioning splint (ARS) for TMJ disk displacement that preserved the patient’s ability to chew by incorporating functional occlusal morphology—something difficult to achieve with traditional wax-up methods [78]. The software allowed the team to customize the splint’s guidance surfaces so that the patient’s posterior teeth still made contact during chewing, illustrating how digitally designed splints can be highly individualized to patient functional needs in ways not easily performed by hand.

Despite these advantages, the CAD phase can be a bottleneck for newcomers. Designing a splint digitally requires familiarity with the software’s tools and an understanding of occlusion. A recent observational study found that dental students took on average 2.6 h to design a mandibular splint on CAD software their first time, and many found the CAD steps challenging [18]. This contrasts with an expert dental technician who might complete a similar design in 15–30 min. Over time, as users gain experience or if partial automation templates are used, the design phase accelerates. In fact, many modern programs offer semi-automated workflows—for instance, 3Shape Splint Studio can auto-generate an initial design with just a few inputs, after which minor edits are made. The learning curve is real, but once mastered, digital design significantly boosts efficiency: adjustments that might require remaking an analog splint can be performed virtually in minutes, and one design can be used to fabricate multiple identical copies if needed (e.g., a patient who wants a backup night guard).

Additionally, virtual articulators in CAD provide a more comprehensive occlusal analysis than a static plaster mounting. Fully adjustable virtual articulator systems can incorporate patient-specific values (condylar inclination, Bennett angle, etc.) to simulate jaw movements on the digital models [79]. This means the clinician can, for example, check how the splint will guide the jaw in lateral excursion or protrusion and adjust the design accordingly. In traditional workflows, such analysis would require complex mechanical articulators and face-bow transfers, which are time-consuming. The digital approach thus improves the accuracy of occlusal customization and can enhance the clinical outcome by reducing errors in the splint’s functional fit.

In summary, the CAD phase allows the clinician/technician to virtually prototype the splint with great control. The result is a design that is optimized for the patient’s dentition and jaw movements. While it demands an initial investment in training and software, the ability to iterate and refine designs on the computer—rather than by repeatedly adjusting acrylic on the patient—is a clear advantage. When the design is finalized and saved, the workflow moves on to manufacturing, typically via 3D printing (additive CAM).

### 4.3. Three-Dimensional Printing and Manufacturing

In the digital workflow, manufacturing of the splint is commonly performed by 3D printing (additive manufacturing). The finalized CAD model (STL file) is imported into the printer’s software, where it is oriented and prepared for printing. In dentistry, the dominant 3D printing technologies for splints are stereolithography (SLA) and digital light processing (DLP), as these vat photopolymerization methods can produce the high resolution and accuracy required for intraoral appliances. A liquid resin specifically made for dental splints (usually a biocompatible methacrylate-based photopolymer) is used. The printer builds the splint layer by layer by curing the resin with a light source (laser or projector), achieving typical layer thicknesses of 50–100 μm or even finer.

Printer setup and orientation: Proper orientation of the splint on the build platform is a critical step that can affect the fit and surface quality. The STL can be positioned flat (0°) or at an angle (often 30–45°) relative to the build platform. Supports are automatically generated to hold the splint in place during printing, especially if it is angled. Research has shown that print orientation has a significant impact on the accuracy and fit of occlusal splints. Vasques and Laganá (2018) [80] tested splints printed at 0°, 30°, and 90° and found the worst fit occurred with 90° (upright) orientation, while 0° yielded the best internal fit in the patients’ mouths [80]. Similarly, an in vitro study by Grymak et al. (2021) noted that splints printed flat (0°) had the highest surface gloss and lowest roughness even before polishing, indicating a very smooth surface, whereas steeper angles introduced more layer steps and roughness [81]. These findings align with the general recommendation to print splints in a somewhat flat orientation if possible to maximize accuracy and minimize the amount of post-processing needed. However, there is a balance to strike: printing completely flat on the build plate can sometimes introduce distortion due to resin shrinkage stresses, and it also means one side of the splint (the intaglio, if printed intaglio-down) is in direct contact with the build plate, emerging very smooth but possibly slightly compressed. Many labs therefore prefer a slight tilt (e.g., 20–30°) which requires supports but allows controlled polymerization and easy resin flow without significantly sacrificing fit [82]. In any case, the orientation is chosen to ensure that critical fit surfaces cure with minimal error, and any supports are placed on non-critical areas (usually on the occlusal surface or edges, which will be polished off later).

Printing process: Once oriented and supported, the job is sent to the 3D printer. The actual print time depends on the printer and the number of splints being produced. A single splint might print in as little as 30–60 min on a fast DLP printer, whereas a batch of multiple splints could take a few hours. One advantage of additive manufacturing is parallel production—multiple splints can be printed in one run if the build platform is large enough, with virtually no increase in total time [35]. This is in contrast to milling, where each splint must be milled individually from a disk. This makes 3D printing particularly suitable for efficient batch production in dental laboratory settings. Indeed, printers are now commonly used in dental labs to fabricate several night guards or orthodontic splints in an overnight build, ready for delivery the next day.

Accuracy of the printed splint is generally high, often within tens of microns of the digital design under optimal settings. For example, one study reported a root-mean-square deviation on the intaglio surface of only ~50 μm when printed at 0° on a calibrated SLA printer [13]. Clinically, this translates to a good initial fit in most cases. Kraemer-Fernández et al. [18] found that about 69% of student-made splints using a digital workflow fit intraorally without need for adjustment at insertion, and the remainder needed only minor grinding (the fit rate likely would be higher with experienced operators) [18]. This suggests that a properly printed splint can often be delivered straight from the printer (after post-processing) with minimal chairside adjustments, whereas traditional acrylic splints almost always require grinding of high spots at delivery due to processing changes.

Advantages over traditional fabrication: The 3D printing step offers major improvements in speed, labor, and material efficiency. Traditional splint fabrication (for a hard acrylic splint) involves flasking and processing PMMA resin or vacuum-forming a thermoplastic sheet over a model, both of which are multi-step, labor-intensive processes. In contrast, once the design is ready, printing is largely automated—the machine does the work while staff can attend to other tasks. Turnaround times are greatly reduced; a splint can be designed and printed on the same day. This has opened the door to chairside splint fabrication in some dental practices. For instance, a dentist with an intraoral scanner and a desktop DLP printer can scan a patient in the morning and deliver a finished night guard by the afternoon or the next day, a level of service that was impractical with conventional methods. Faster delivery not only improves patient satisfaction but also allows protection (for bruxism cases, etc.) to start sooner.

Beyond speed and precision, the decision to fabricate splints chairside or via a laboratory model depends on several factors, including equipment availability, training level, and case complexity. Table 3 provides a comparative overview of the two workflows, highlighting clinical scenarios where one may be preferable to the other.

In-house (chairside) production allows rapid turnaround and complete customization, which is particularly advantageous for TMD splints or when iterative adjustments are needed. However, it requires significant investment and staff training. In contrast, outsourcing to a dental lab ensures high consistency and is ideal for complex or multi-unit cases, though with slightly longer delivery times [10,17,24,83,84].

In terms of material properties, milled splints are still considered the gold standard for strength because they are made from industrially polymerized PMMA blocks. Three-dimensionally printed resins, being light-cured, can be slightly less cross-linked and may exhibit lower flexural strength and long-term durability [1]. Milled appliances have virtually no internal porosity and have excellent density, whereas printed ones might have minor layer interfaces. However, ongoing advancements in resin chemistry (e.g., new monomers and hybrid materials) are closing this gap. Many 3D printing splint resins are now FDA-cleared/CE-marked and have flexural strength and hardness approaching that of conventional acrylics. Van Lingen and Tribst [83] note that while milled splints tend to have superior longevity, 3D-printed splints offer greater flexibility and a faster turnaround, making them highly suitable for many clinical scenarios—especially when rapid provision or iterative prototyping of splints is needed [83]. In practice, many dentists reserve milled splints for heavy bruxers or long-term use and are increasingly using printed splints for the majority of cases due to their convenience. Importantly, initial studies indicate that patients adapt equally well to printed splints, and clinical outcomes (in terms of symptom relief for TMD or protection of teeth) are comparable to traditional splints as long as the device is appropriately designed [17]. A forthcoming randomized trial is set to provide high-level evidence by comparing printed and milled splints in a crossover design; the expectation (and hypothesis) is that printed splints will not be inferior in clinical efficacy while offering advantages in fabrication efficiency [17].

By the end of the printing phase, the result is a raw printed splint in a translucent (or transparent) resin, with support structures attached if used. This raw product must undergo post-processing before it is ready for patient use. Notably, the digital workflow up to this point (scanning → CAD → printing) is vastly more streamlined and faster than the traditional workflow (impression → plaster model → wax-up → flasking/pressing acrylic or vacuum form → trimming). One publication succinctly stated that the digital workflow is more time-efficient and yields a better fit and patient comfort than the conventional workflow of alginate impressions, stone casts, and heat-cured acrylic fabrication [17]. The next subsection addresses the final steps needed to turn the printed piece into a polished, clinic-ready appliance.

**Table 3:** Comparison of Chairside vs. Laboratory-Based Digital Workflows for 3D-Printed Splints.

Laboratory-Based Workflow	Chairside Workflow	Aspect
Same as chairside; files are exported and sent to the lab [74]	Performed in-clinic using IOS by dentist/staff [2,73,74,84]	Scanning
By a trained dental technician using advanced design tools [79]	In-house design by clinician (requires training) [76,78,85]	CAD
Often included in the lab’s internal workflow—cost embedded in per-unit fee [80]	Requires full CAD license (e.g., 3Shape Splint Studio, Exocad) [76]	Software Needs
Uses industrial or high-capacity 3D printers with optimized calibration [80]	Requires in-office 3D printer + curing unit (SLA/DLP preferred) [2,78]	Printing Equipment
Typically 2–7 working days, depending on lab and shipping	Same-day or next-day delivery possible [2,86]	Turnaround Time
Requires communication and possible redesign turnaround with the lab	Direct control over design iterations and reprints [76,77]	Customization Control
No hardware investment needed; cost per case basis	High initial investment in scanner, printer, post-processing units, software licenses [87]	Cost of Setup
Minimal clinic-side effort; handled entirely by trained lab technicians	Significant training is required in scanning, design, and post-processing [73]	Staff Training
Best for complex prosthetics, batch production, or long-term splints requiring extra finishing [88,89]	Ideal for emergency/night guards, TMD splints, or when fast iteration is needed [2,76,77]	Best Use Cases
High reproducibility ensured by lab QA protocols and calibration systems [80]	Dependent on clinician/operator skill and printer maintenance [74,83]	Quality Consistency

Abbreviations: IOS—intraoral scanner; TMD—temporomandibular disorders. References: [2,73,74,76,77,78,79,80,83,84,85,86,87,88,89].

### 4.4. Post-Processing and Finishing

After printing, the splint must be post-processed to achieve its final mechanical and biological properties. This phase includes cleaning, curing, support removal, and polishing—each essential for safety, fit, and patient comfort.

As described in Section 3.3 and summarized in Table 1, post-processing steps such as IPA cleaning, UV curing, and nitrogen polymerization enhance polymer conversion, reduce residual monomers, and improve flexural strength and biocompatibility [58,62,90,91,92,93,94]. Support removal is typically performed with rotary tools or polishing wheels, taking care not to over-polish fit-critical surfaces [92].

Polishing improves comfort and hygiene by reducing surface roughness. Grymak et al. (2021) found that splints printed flat (0° orientation) exhibited very low roughness (~0.06 µm), requiring minimal polishing, while steeper print angles benefited significantly from mechanical finishing [81]. Polishing generally proceeds in two steps—initial smoothing with pumice, followed by high-gloss finishing paste. Most clinicians polish all surfaces regardless of orientation to ensure a smooth feel and reduce tissue irritation.

Surface texture has important clinical implications. Smoother splints exhibit less bacterial adhesion and biofilm accumulation, reducing odor, staining, and hygiene concerns. In vitro studies confirm that printed resin surfaces, when well-polished, show lower biofilm mass than conventional PMMA or thermoplastic materials [95]. Resin formulation also affects outcomes; one softer resin with higher surface roughness accumulated more plaque, emphasizing the importance of both polishing and material selection.

After polishing, the splint is tried intraorally for final assessment. With well-executed digital workflows, minimal or no chairside adjustments are typically needed. In one study, even novice designers achieved accurate fit in ~66% of cases without adjustments, underscoring the precision of digital design and printing [18]. Another protocol projects improved comfort and fit for digital splints compared to handmade versions [17].

The splint is then disinfected and delivered along with patient instructions. Digital scan and design files are preserved, enabling easy reproduction in case of breakage or loss—an advantage rarely feasible with traditional methods [17].

In summary, when each step of the digital workflow is properly executed—from scanning and CAD to post-processing—the result is a clinically effective, hygienic, and patient-friendly splint. Digital methods not only match conventional standards but may exceed them in speed, comfort, and reproducibility [17,95].

## 5. Clinical Performance and Outcomes of 3D-Printed Splints

### 5.1. Occlusal Splints (TMD and Bruxism)

3D-printed occlusal splints have been extensively evaluated both in vitro and clinically. Barbur et al. reported good accuracy, hardness, and flexural strength in splints fabricated from a biocompatible resin [24]. While many studies indicate 3D-printed splints perform well mechanically, there are some conflicting findings. Prpić et al. observed higher flexural strength in conventionally heat-cured splints than in printed ones [1]. In terms of wear, Abad-Coronel et al. noted that milled splints resisted wear better than 3D-printed resin [96]. Importantly, the long-term stability of printed splints has come under scrutiny: an artificial aging study showed that aging can significantly reduce the compressive modulus and strength of printed splint material, though proper post-processing (like additional polishing and curing) can mitigate these effects [97]. On the whole, the literature suggests that with the appropriate resin and printing technology, 3D-printed occlusal splints achieve the necessary strength and durability for clinical use—acknowledging that material choice and processing conditions are crucial determinants of performance.

Biocompatibility studies also support the safety of 3D-printed splint materials. Bürgers et al. [98] directly compared printed splint resin to traditional acrylic and milled resin in vitro. They found no significant differences in cytotoxic effects among the manufacturing methods—all materials showed cell viability above critical thresholds, indicating that 3D-printed resins (when properly post-cured) are as biocompatible as conventional splint materials. This aligns with other reports confirming that modern printable dental resins elicit minimal cytotoxicity and are safe for intraoral use. Thus, from a materials perspective, 3D-printed occlusal splints meet the mechanical and biological requirements for splint therapy, though clinicians should be aware of resin-specific aging behavior and follow manufacturers’ curing instructions to ensure longevity.

Clinically, 3D-printed occlusal splints demonstrate comparable—and in some aspects improved—outcomes relative to conventionally fabricated splints. A recent randomized crossover trial evaluated bruxism patients over 3 months. They reported that both printed and traditional (hand-fabricated) splints were effective in reducing parafunctional muscle activity, with no significant difference in the overall sleep bruxism index between the groups [18]. Notably, patients using 3D-printed splints reported greater subjective improvement, with 64% perceiving lifestyle benefits compared to a lower percentage in the conventional group [18]. Such initial comfort gains may partly reflect novelty or placebo effects, underscoring the need for longer-term objective measures (e.g., EMG data, quantification of tooth wear, and frequency of splint adjustments). Many patients found the printed splints more comfortable, which the authors attributed to the digital design’s precision and the printed resin’s more uniform material properties. Indeed, the printed splints showed a more uniform elasticity across the occlusal surface, potentially leading to more even occlusal force distribution [18]. This enhanced comfort and adaptation may explain why, despite similar objective efficacy, patient satisfaction tended to favor 3D-printed appliances in the short term. Another study on temporomandibular disorder (TMD) patients compared digitally manufactured splints to traditional repositioning splints over 6+ months. Both methods improved pain and jaw function, but the 3D-printed splint group showed greater pain relief and mouth-opening increases in early follow-ups [99]. By one month, the printed splints achieved an 87.3% effective rate (pain reduction), slightly outperforming the 82.9% rate of the conventional group [99]. The authors noted that the digital workflow (intraoral scanning, virtual jaw relation recording, and CAD) eliminated many manual steps and errors, resulting in more accurate splint fit and presumably better therapeutic outcomes [99]. These clinical findings suggest that 3D-printed occlusal splints are at least non-inferior to conventional ones in managing bruxism and TMD, with potential advantages in patient-reported comfort and faster adaptation. Ongoing trials are further investigating long-term outcomes: for example, Rabel et al. have a 2024 crossover trial in progress to formally test the non-inferiority of 3D-printed versus milled stabilization splints in bruxism/TMD patients, measuring quality of life, satisfaction, pain relief, and device longevity [17]. The expectation is that printed splints will perform equivalently to the gold-standard milled acrylic devices, which would validate 3D printing as a routine fabrication method in occlusal therapy. From a cost-effectiveness standpoint, while chairside 3D printing offers rapid turnaround and lower per-unit labor costs, milled PMMA splints may prove more durable and economical over the long term in patients with high wear risk.

### 5.2. Orthodontic Splints (Aligners and Retainers)

Additive manufacturing has also expanded into orthodontic appliances, including clear aligner trays and retainers, which can be viewed as types of splints guiding tooth movement. Traditional clear aligners are made by thermoforming plastic over 3D-printed molds, but recent advances enable direct 3D printing of aligners from biocompatible clear resins [100,101]. According to Torkomian et al. [100], directly printed aligners exhibit mechanical and physical properties on par with conventional thermoformed aligners. Their review found that both types show sufficient strength and elasticity for orthodontic forces, but 3D-printed aligners can have unique advantages such as shape memory behavior and greater design flexibility in thickness and geometry [102]. For instance, shape memory polymers used in some printed aligners allow the appliance to consistently return to its programmed shape, potentially providing a more stable force over time compared to thermoformed plastics, which can deform. Printed aligners also demonstrated high dimensional accuracy (trueness and precision), often surpassing that of vacuum-formed trays [102]. However, current studies note that factors like layer thickness and print orientation can introduce minor thickness variations, so optimization of print settings is necessary to fully capitalize on the accuracy gains [102]. Overall, the systematic review concluded that 3D-printed aligners are a promising alternative, likely to become clinically viable in the near future, though more in vivo research is needed to confirm their performance in active tooth movement [102].

Initial clinical and laboratory evaluations support these findings. A scoping review by Jungbauer et al. [103] surveyed the recent literature and reported that directly printed aligners can deliver the intended forces for tooth movement with comparable effectiveness to conventional aligners while reducing fabrication time and waste. Additionally, printing technology allows for complex features like integrated attachments or variable thickness zones in aligners that are difficult to achieve with thermoforming [103]. In vitro force measurements (e.g., on simulated tooth models) have shown that printed aligners maintain more consistent force levels during deformation, whereas thermoformed aligners may lose force as the plastic relaxes [103]. Another study found that a shape-memory resin aligner exerted predictable forces and better control over tooth movements due to its homogeneous material properties [104]. For orthodontic retainers, 3D printing is also being explored as a direct fabrication method. Firlej et al. [105] evaluated 3D-printed retainer materials and found tensile strength and elasticity comparable to conventional retainer plastics, suggesting printed retainers could be similarly durable. Importantly, a biocompatibility comparison of aligner materials indicated that 3D-printed aligner resins did not produce higher cytotoxicity or adverse cell responses than standard thermoplastic aligner sheets [106]. However, a study by Willi et al. [107] quantitatively assessed the degree of conversion and the leaching of compounds from 3D-printed aligners made of photopolymerized resin (Tera Harz TC85A), finding no detectable bisphenol A (BPA) but variable amounts of urethane dimethacrylate (UDMA) monomer, ranging from 29 to 96 µg/l, raising concerns about potential health hazards after repeated intraoral exposure.

Clinical outcome data for fully 3D-printed aligner systems are still limited but emerging. Early pilot studies report that treatment results with printed aligners (in terms of tooth movement achieved vs. planned) are on the order of 80–85% accuracy, which is similar to conventional aligner outcomes in those small samples. Patients have so far tolerated printed aligners well, with fit and comfort rated as good as the traditional trays in initial trials (e.g., no significant difference in aligner-induced discomfort or speech issues). Moreover, digital workflows enable rapid fabrication of replacement aligners or adjustments: a lost or cracked retainer can be reprinted from the saved design within hours, an advantage over laborious remolding. In summary, while the long-term clinical effectiveness of 3D-printed orthodontic splints (aligners/retainers) is still being validated, current evidence suggests they can match conventional devices in biomechanical function [102]. The main benefits lie in efficiency and customization—and as materials continue to improve, we can expect printed aligners to become a routine option in orthodontic practice, providing equally successful tooth alignment outcomes [102].

### 5.3. Surgical Splints (Orthognathic and Implant Guides)

In surgical dentistry, 3D printing has revolutionized the fabrication of splints and guides for procedures like orthognathic surgery and implant placement. Digital planning allows the creation of precise surgical splints that transfer the virtual treatment plan to the patient with high fidelity. Research overwhelmingly shows that 3D-printed orthognathic splints achieve accuracy equivalent to conventional acrylic splints while streamlining the workflow. For example, Sarkarat et al. [32] compared intermediate splints made by traditional manual fabrication versus CAD-designed 3D printing in a cohort of 10 jaw surgery patients. They reported excellent agreement between printed and conventional splints—linear measurements of maxillary movement differed by a mere 0.0–0.3 mm on average, with intra-class correlation > 0.9, indicating virtually identical positioning outcomes. Moreover, after conducting the actual surgeries using the 3D-printed splints, postoperative skeletal positions closely matched the presurgical digital plan, with no significant differences between planned and achieved cephalometric values [32]. This confirms that printed guides can accurately translate surgical plans to reality. Similarly, a systematic review by Rajagopalan et al. [108] found that splints produced by 3D printing were as accurate in achieving jaw movements as traditional splints, with mean linear discrepancies often well under 1 mm [108]. Surgeons also report reduced time adjusting splints intraoperatively when using printed versions, since the fit on dental arches is more precise from the get-go.

Notably, when comparing different fabrication methods for surgical splints (handmade vs. milled vs. printed), studies have found only minor differences in accuracy and fit. In an in vitro experiment, Lee et al. [109], one group fabricated identical splint designs three ways: subtractive milling, SLA printing, and DLP printing. The milled splints showed the lowest mean deviation from the digital design (~0.11 mm), slightly better than DLP (~0.14 mm) and SLA (~0.16 mm) printed splints. However, all methods were well within clinically acceptable accuracy, and interestingly, the DLP-printed splints had the best seating fit on the dental models compared to others [109]. The authors concluded that while milled polycarbonate splints might offer marginally higher trueness, the difference is likely not clinically significant, and both printed and milled splints provided excellent fit and stability during mock surgery. In practice, the slight trade-off in accuracy for printed splints is offset by practical advantages: 3D printing is generally faster, less labor-intensive, and allows fabrication of complex integrated designs (for instance, splints that double as cutting guides). There have been few reports of failures of 3D-printed surgical splints; on the contrary, their consistency and reproducibility are cited as strengths. One consideration is that the resin splints can be more brittle than acrylic—so care must be taken during surgery to avoid dropping or overly bending a printed splint. However, when needed, a broken splint can be quickly reprinted. Also, sterilization protocols (such as low-temperature hydrogen peroxide gas plasma) are used since standard autoclaving could deform some printed resins; studies like Török et al. [110] have shown that proper sterilization does not significantly distort printed guides, preserving accuracy for the operating room.

For implant surgery, 3D-printed guides (splints that fit on teeth or mucosa to direct implant drills) have become state-of-the-art due to their impact on surgical outcomes. Numerous studies have documented that static computer-aided implant guides fabricated by 3D printing yield high placement accuracy. Lo Russo et al. [111] analyzed both milled and printed guide accuracy across multiple trials. They found that the deviation between planned and actual implant positions was low for both methods (typically around 1 mm at the implant apex and 2–4 degrees angulation). The manufacturing technique (milling versus printing) had a minimal effect on accuracy, confirming that printed guides can achieve the same precision as traditionally milled guides in transferring the implant plan to the patient’s mouth. Another study that compared four different 3D printers reported all printed guides were dimensionally accurate, with only ~0.1–0.2 mm differences among printer types [112]. Clinically, the use of these guides (as opposed to freehand surgery) significantly improves implant placement accuracy and reduces the risk of angular deviations or damage to vital structures [113]. For instance, fully guided immediate implant placement with a printed guide was able to consistently achieve implant positioning within 1–2° of the plan and had high success rates at one-year follow-up [114]. The consensus is that for implant and orthognathic applications, 3D-printed splints perform equivalently to conventional methods in terms of surgical accuracy and clinical effectiveness. They also provide workflow efficiencies (fewer analog model steps, reduced adjustment) and enable advanced procedures—surgeons can treat complex cases with confidence using digitally designed splints that precisely custom-fit the patient.

### 5.4. Restorative and Endodontic Splints

Beyond occlusion and surgery, 3D printing has been applied to splints and guides in restorative dentistry and endodontics, yielding notable improvements in clinical outcomes. In endodontics, specially designed 3D-printed guides (sometimes called endodontic splints or stents) are used to locate calcified root canals or to guide periapical surgery. Studies have shown drastic gains in accuracy and efficacy with these devices. Connert et al. [115] demonstrated that guided endodontic access in teeth with calcified canals was far superior to the freehand approach. In their experiment with simulated calcified incisors, the success rate of locating the canal went from ~42% with traditional techniques to 92% with a 3D-printed guide [115]. The guided method also preserved far more tooth structure—only about 10 mm3 of dentin removed on average, versus 50 mm3 removed with conventional drilling—and halved the procedure time (11 min vs. 22 min) regardless of the operator’s experience [115]. These findings confirm that 3D-printed guides allow a minimally invasive tunnel to be drilled directly to the canal, avoiding the excessive dentin removal and frustration often encountered in calcified cases. Clinically, this translates to higher success in treating “impossible” calcified canals, with fewer perforations or missed canals [116]. Multiple case reports and reviews have reinforced that guided endodontics improves precision, conserves tooth structure, and can even enable treatment of teeth that would otherwise be considered untreatable by conventional means [117,118]. Likewise, in endodontic surgery (apicoectomy), printed guides help in achieving the correct osteotomy location and angle. Ackerman et al. [119] found that using a CBCT-planned 3D-printed guide to target the root apex resulted in significantly smaller deviations from the ideal target compared to freehand surgery. In their cadaver trial, all guided osteotomies successfully accessed the intended root apices, whereas nearly half of freehand attempts missed the target area [119]. This improved accuracy directly correlates with better surgical outcomes and healing, since the periapical pathology can be predictably removed with minimal bone removal. No notable adverse events (such as guide failure or toxicity) were reported with the use of these resin guides.

In restorative and prosthodontic domains, 3D-printed “splints” or guides are employed in various ways to enhance treatment precision. For instance, in the placement of dental veneers or crowns, clinicians have used 3D-printed index splints derived from digital wax-ups to guide tooth preparation or to verify reduction uniformity [120]. These splints, analogous to a surgical guide, ensure that minimal necessary tooth structure is removed and the planned restoration contours are achieved [121]. While formal studies are scarce, initial reports suggest these printed prep guides improve the accuracy of extensive rehabilitations and reduce chair time for adjustments. Another emerging application is in prosthodontic rehabilitation of edentulous patients: printed therapeutic splints can be used as trial dentures or interim prostheses to test the vertical dimension and occlusal scheme before definitive denture fabrication. Because they are quick to produce, several iterations can be tried if needed, leading to a better final outcome. Additionally, periodontal splints (used to stabilize mobile teeth) have traditionally been made from metal or fiber splints customized chairside; now, digital intraoral scans can facilitate the design of a rigid splint that 3D prints to precisely fit the lingual surfaces of the involved teeth for bonding. Although robust clinical data on printed periodontal splints are not yet published, this approach has been piloted with promising results in terms of fit and patient comfort.

In summary, across restorative and endodontic applications, 3D-printed guides and splints are proving to enhance precision and efficacy. They allow clinicians to execute procedures with a level of accuracy difficult to attain freehand—whether it is locating a hidden root canal or preparing multiple teeth to exact dimensions. Patient outcomes benefit as treatment becomes more conservative (preserving healthy structures) and predictable. Long-term reports are still limited in these domains, but early success and high adoption rates indicate that 3D-printed splints will continue to expand in use. So far, failure or complication rates appear very low; the main limitation is the need for meticulous digital planning. As more practitioners become comfortable with CAD workflows, the clinical performance of 3D-printed splints in dentistry underscores a broad positive trend: they either match or improve upon conventionally fabricated devices in most metrics—from mechanical strength and accuracy to patient comfort and satisfaction—marking a significant advancement in dental care through digital fabrication technology.

## 6. Benefits, Limitations, and Clinical Recommendations

3D-printed dental splints have introduced a range of practical advantages over conventional fabrication methods, alongside certain limitations that clinicians must consider. An evidence-based understanding of these pros and cons is essential to maximize clinical success. Below, we summarize key benefits and challenges of 3D-printed splints, followed by clear clinical recommendations for general practice and specialty use.

### 6.1. Benefits and Advantages

- Customization and precision: Digital design and 3D printing enable highly personalized splints that precisely match patient anatomy, improving fit and comfort. The technology allows complex geometries and fine details that are difficult to achieve with traditional methods. Studies have noted that 3D-printed splints can be produced with excellent accuracy and patient-specific details, enhancing overall comfort and therapeutic effectiveness [13,83]. Printed splints often exhibit comparable accuracy to milled splints and superior fit to purely manual techniques, provided that proper printing orientation is chosen during fabrication [13,83].
- Rapid digital workflow: 3D printing significantly accelerates splint production by streamlining the workflow from intraoral scanning to appliance delivery [121]. Once a digital model is obtained, splints can be fabricated within hours, reducing turnaround time compared to lab-fabricated acrylic splints. This faster production is particularly evident when multiple splints are printed in a single batch. A narrative review highlighted that 3D printing offers “faster turnaround times” than traditional methods [19,83]. In clinical settings, in-house printing can allow delivery of a splint on the same day or next visit, which is highly convenient for both patient and practitioner.
- Reproducibility and digital archiving: The digital nature of the process means that once a splint is designed, it can be saved and reproduced exactly [13,83]. If a patient loses or breaks their appliance, an identical replacement can be printed without needing new impressions or redesign. This reproducibility ensures consistency in treatment; by contrast, manually fabricated splints can have minor variability each time. Moreover, digital models obviate the need to store physical casts, reducing storage requirements in the clinic.
- Reduced material waste: Additive manufacturing is efficient in material usage, since splints are built layer-by-layer with minimal excess. Unlike milling (subtractive manufacturing), which carves from a puck and wastes the cut-away material, 3D printing uses only the resin needed for the device. A recent scoping review of 3D printing in dentistry noted the ability to produce complex structures with less material waste as a major advantage [83,121]. Environmentally and economically, this efficiency is beneficial over time.
- Patient comfort: Clinical reports suggest 3D-printed splints often have a more uniform internal fit and smoother surfaces, improving patient comfort during wear [23]. The digital design can incorporate evenly distributed thickness and well-polished occlusal surfaces. In a short-term trial, the regular surface of printed splints was associated with more stable jaw muscle activity during sleep bruxism, indicating a therapeutic comfort benefit [19,83]. Improved comfort can translate to better patient compliance in wearing the splint as prescribed.

### 6.2. Limitations and Challenges

Mechanical strength and durability: One well-documented limitation of printed splints has been their mechanical properties relative to conventional materials. Milled splints made from poly (methyl methacrylate) (PMMA) disks are known for superior strength and longevity, whereas some 3D-printed resins can be more brittle or wear faster [1,68]. In an in vitro study, milled PMMA appliances showed significantly higher flexural strength and wear resistance than several printed resin splints [1,68]. Printed splints, especially older generations of resin, may be prone to edge chipping or surface wear over time under heavy occlusal loads [109]. However, it should be noted that material science is advancing: newer high-impact printable resins (e.g., cross-linked dimethacrylates and “flexible” resin formulations) have demonstrated improved strength and wear characteristics approaching those of milled PMMA [94]. Nonetheless, long-term clinical data are still limited on whether these materials can match the multi-year service life of traditional splints.

- Material aging and stability: The oral environment tests the long-term stability of any splint material through cyclic loading, thermal changes, and moisture. Resin-based 3D-printed splints can undergo hydrolytic and thermal aging that may reduce mechanical performance over time. Laboratory aging simulations have shown that certain printed splint materials experience a decline in hardness and strength after prolonged moisture and thermal cycling [122]. For example, one study found a significant reduction in tensile strength and hardness of some printed resins after six months of simulated use, although the values remained clinically acceptable [122]. This suggests that while printed splints should function well in the short-to-medium term, their properties may change with extended use, and periodic monitoring or replacement may be necessary for long-term cases.
- Post-processing requirements: Unlike a finished milled splint that emerges ready for polishing, a printed splint requires several post-printing steps. These include cleaning (typically in isopropyl alcohol to remove uncured resin) and mandatory post-curing under UV light to fully polymerize the resin. Improper or insufficient post-curing can leave residual monomers that are both weaker and potentially cytotoxic to oral tissues. The need for careful post-processing means the workflow is technique-sensitive—operators must adhere to manufacturer protocols for curing time, light intensity, and washing to ensure the final appliance is safe and durable. Additional finishing may be required to remove support nibs and smooth the surface. This adds labor and potential for error; one review cites post-processing demands as a current hurdle to efficiency [93]. In summary, while printing itself is automated, the downstream steps must be well managed in the dental office or lab.
- Equipment and material costs: The initial investment in a 3D printing ecosystem can be high for a dental practice. Costs include the printer hardware, ancillary devices (washer, curing light units), and ongoing materials and maintenance. High-quality biocompatible resin cartridges can be expensive, and printers require calibration and occasional part replacement. A 2025 industry review identified high upfront costs and the expense of proprietary materials as factors that “hinder…broader adoption” of 3D printing in dentistry [123]. However, it is worth noting that the per-unit cost of splints can become quite low once the system is in place (often only a few dollars of resin per splint), especially if many devices are produced. Thus, cost concerns are more pronounced for low-volume settings or early adopters. Over time, as printer prices decrease and material options broaden, cost-effectiveness is expected to improve.
- Biocompatibility considerations: All dental appliances must be safe for intraoral use. The photopolymer resins used for printing splints are formulated to be biocompatible, and recent evidence is reassuring. A 2024 cytotoxicity study comparing 3D-printed splint resin to conventional acrylics found that the printed resin (after proper curing) induced no greater cell toxicity than heat-cured or vacuum-formed materials, meeting ISO standards [98]. Nevertheless, any uncured resin or solvent residue can irritate tissues, so meticulous processing is critical. Some patients report a slight resin odor or taste initially, which usually dissipates after thorough cleaning. Overall, while biocompatibility of well-processed printed splints is high, dentists must ensure proper fabrication procedures to avoid adverse reactions.

### 6.3. Clinical Recommendations

In light of the above benefits and limitations, the following evidence-based recommendations can be made for clinicians using 3D-printed splints:

- Dentists and laboratory technicians should pursue training in digital scanning, CAD, and printer operation before incorporating 3D-printed splints into practice. A skilled workflow ensures that the advantages of customization and speed are realized without compromising fit or safety. Close attention should be paid to manufacturer guidelines for printer calibration and resin handling.
- 3D-printed splints are suitable for the majority of occlusal guard indications (e.g., bruxism night guards, stabilizing splints for temporomandibular disorders, etc.), offering comparable therapeutic outcomes to conventional splints in short-term studies [17,121]. For patients who require rapid turnaround or those who cannot tolerate traditional impressions (such as a strong gag reflex or traumatically loosened teeth), printed splints are especially advantageous. However, in cases of extremely high occlusal forces (e.g., severe bruxers) or where an appliance will be used for many years without replacement, practitioners may consider using a milled or conventional splint.
- Only FDA-approved (or equivalent) dental splint resins should be used for intraoral appliances. These resins have been formulated and tested for biocompatibility and mechanical performance. Emerging “soft” or flexible splint resins can be considered for patients who find hard acrylic appliances uncomfortable—such materials have shown promising strength and wear resistance despite their flexibility [124]. Clinicians should stay updated on material advances, as the resin landscape is quickly evolving.
- How a splint is oriented in the printer can influence its fit and surface quality. It is recommended to follow guidelines (often provided by resin or printer manufacturers) for orientation—for example, printing at a slight angle can reduce the appearance of layer striations on the intaglio surface and improve fit accuracy [13,125]. During the CAD phase, ensure adequate thickness (at least ~1.5–2.0 mm in occlusal areas) and smooth transitions, as very thin or sharp areas are more prone to fracture in printed resin. Incorporating full-arch coverage and reinforcing any protruding sections in the design will enhance longevity.
- After printing, the splint must be thoroughly washed to remove uncured resin and then cured under the proper light and time conditions. It is recommended to use an automated wash unit and a calibrated curing light specific to the resin wavelength. Any support attachments should be carefully removed, and the splint polished. This ensures the appliance reaches its intended strength and is free of irritants. The finished splint should be inspected for complete curing (no tacky surfaces) and rinsed before delivery. Following these steps will yield a device that is safe (nontoxic) and durable in the mouth [98].
- When delivering a 3D-printed splint, advise the patient on proper care (regular cleaning with mild soap and cool water, avoiding high heat). Schedule follow-ups to check the splint’s fit and integrity, for example, at 6 months and annually. Early data suggest printed splints can function well for at least 6–12 months under normal use without significant wear [99]. If the splint shows signs of material fatigue (cracks, loss of fit) or if the patient’s dental conditions change, the digital file makes it straightforward to print a fresh appliance. This proactive approach will ensure the patient continues to receive effective protection.
- Specialists should integrate 3D-printed splints into relevant treatments. For instance, in pediatric dentistry and trauma cases, a digitally fabricated splint can stabilize injured teeth with less risk than wire-composite methods. A recent clinical study in children with dental trauma found that CAD/CAM-designed 3D-printed splint therapy achieved equivalent healing outcomes to conventional splints while minimizing iatrogenic tooth movement during impression-taking [126]. Orthodontists can use 3D printing to create interim splints or retainers with precise fit, and oral surgeons can fabricate surgical splints or stents for orthognathic cases more efficiently. In general practice, the use of 3D-printed night guards and occlusal devices is becoming a standard option, and practitioners should feel confident that when properly made, these splints will perform on par with traditional appliances in protecting the dentition and TMJ.

By understanding the benefits, addressing the limitations, and following best-practice protocols, clinicians can fully leverage 3D printing technology to provide effective and efficient splint therapy across various dental disciplines.

## 7. Future Trends and Research Directions

The landscape of 3D-printed splints is dynamic, with ongoing research and innovation poised to further enhance their clinical performance and expand their applications. As digital dentistry progresses, we anticipate smarter materials, more automated design processes, and refined manufacturing techniques that will shape the next generation of dental splints. Equally important, current research gaps are being recognized, pointing to areas where further investigation is needed. This section discusses emerging trends and outlines future directions for research.

### 7.1. Smart Materials and Multi-Material Printing

One exciting frontier is the development of smart materials for splint fabrication. Smart materials can respond to environmental stimuli or possess shape-memory properties, opening the door to splints that adapt intraorally over time. For example, researchers are exploring 4D printing—essentially 3D printing with time as the fourth dimension—whereby a splint could be printed in a certain shape and then later morph or self-adjust in response to mouth temperature or pH changes [127,128]. Although still largely experimental in dentistry, 4D-printed appliances might one day provide dynamic fitting, such as automatically tightening a splint as edema subsides or delivering phased orthodontic forces. A recent narrative review highlighted that 4D printing, achieved through advanced multi-material printers or stimuli-responsive polymers, could produce dental devices that change their morphology in response to stimuli (e.g., heat or moisture) [128]. Such technology could be particularly useful for pediatric or orthognathic surgical splints, where anatomical conditions evolve.

In the nearer term, multi-material 3D printing is becoming feasible and may greatly improve splint function. Current desktop 3D printers typically use a single material at a time, but high-end systems (such as PolyJet triple-jetting technology) can print different materials in one object [129]. This means a splint could be manufactured with, for instance, a rigid framework for strength and a soft liner for cushioning the teeth and gums. Early work in this area is promising: a 2023 study using triple-jetting (PolyJet) printing demonstrated that a multi-material resin splint had mechanical properties on par with conventional PMMA, combining a stiff and flexible resin in one build [24]. The authors reported favorable flexural and tensile strength in the 3D-printed splints and suggested this approach could “replace conventional heat-cured resin” in the future [24]. We anticipate more widespread use of multi-material printers in dentistry, which will allow truly hybrid splints that optimize comfort without sacrificing durability—an appliance with a hard occlusal surface for wear resistance and a soft internal surface for tooth interface could markedly improve patient experience.

Another material-related trend is the refinement of resin chemistry. Manufacturers are introducing next-generation splint resins that are tougher, more wear-resistant, and less brittle. Some resins incorporate nanoparticles or reinforcing fibers to boost strength. Others, like flexible splint materials, aim to absorb forces and reduce the risk of fracture. Notably, one in vitro evaluation found that a popular flexible resin (KeySplint Soft) actually exhibited the highest fracture toughness and wear resistance among tested splint materials, outperforming even harder resins [130]. These advancements suggest that future splints might simultaneously become stronger and softer, tailoring material properties in ways not previously possible with monolithic acrylic. Ongoing materials science research is likely to yield splint polymers with improved longevity (e.g., better resistance to salivary absorption and aging) and enhanced esthetics (clearer, less yellowing over time), addressing some current limitations.

### 7.2. AI-Driven Splint Design and Automation

Artificial intelligence (AI) and machine learning are rapidly being integrated into dental workflows, and splint design is no exception. Traditionally, designing a custom occlusal splint in CAD software requires manual input and expertise to define the occlusal scheme, guidance ramps, and contact distribution. Now, AI-based design software can automate much of this process. Recently launched dental AI platforms can generate a complete splint design from a digital impression and a few clinician inputs (like desired jaw relationship) in a matter of minutes. For example, cloud-based services from major dental companies allow practitioners to upload a patient’s scans and receive an AI-designed night guard ready for 3D printing. This dramatically speeds up the design phase, making same-day splint fabrication more practical. A clinical technique report in 2024 described using two different AI-powered software programs (Medit Splints and 3Shape Automate) to automatically design occlusal devices, which were then additively manufactured [131]. The authors noted that AI design was able to perform in seconds what used to take a technician hours, consistently producing functional designs that required minimal adjustment. As this technology matures, we can expect AI to handle complex aspects like ideal occlusal contact placement and equilibration of the splint, guided by large datasets of successful cases.

Beyond design, AI can assist in predictive modeling and customization. Machine learning algorithms might analyze a patient’s orthodontic or prosthetic data (e.g., bite force distribution, tooth wear patterns) to suggest an optimal splint thickness or reinforcement in specific areas. AI could also be used to predict which patients will benefit most from a particular splint material or design, personalizing the treatment planning. Research is underway comparing AI-designed splints with those made by human technicians; early results indicate that AI models achieve comparable occlusal fit and require fewer adjustments chairside [132]. In the near future, we may see fully automated end-to-end workflows: a digital scan is taken, an AI algorithm designs the splint almost instantaneously, and a 3D printer produces it within the same appointment. Such integration could revolutionize efficiency in delivering occlusal guards and other appliances.

### 7.3. Chairside Printing and Workflow Improvements

The drive for faster, more convenient fabrication is leading to innovations in printer technology specifically geared for chairside use. Traditional SLA/DLP printers, while accurate, often take a couple of hours to print a single splint and then additional time for post-curing. New printer designs and resin formulations are drastically cutting these times. For instance, some printers now employ high-speed LCD or layered projection systems that can print multiple splints in under 30 min by optimizing light exposure and layer curing times. Concurrently, immediate curing concepts are being explored, such as resin that cures to a functional state on-the-fly or high-intensity curing units that shorten post-cure to mere minutes. These advances mean that a patient could conceivably have a scan and receive a finished splint within a single hour-long visit, which is a game-changer for practices offering same-day services.

Another improvement is in printer reliability and user-friendliness. Next-generation dental 3D printers are being designed as turnkey appliances with pre-calibrated settings, printable resin cartridges, and software that automates support generation and positioning. This reduces the learning curve and potential errors for dental staff. Many systems now integrate with dental CAD software and even with intraoral scanners to create a seamless digital system. As an example, a practitioner can scan a patient, and with minimal clicks, send the file to print with an AI-optimized orientation and support setup. These streamlined workflows are supported by dental companies providing validated “splint printing” workflows (including specific printers, resins, and curing units that are tested together). Such validation ensures that the printed splint will have the claimed accuracy and strength, addressing variability issues.

Importantly, the cost barrier of 3D printing is expected to decrease as the technology becomes more widespread. Already, open-source and lower-cost printers are entering the market, and resin prices may drop with competition and bulk use. Chairside printing will become economically attractive to smaller clinics as ROI (“return on investment”) improves. Combined with the time savings from AI design and faster printers, 3D-printed splints are likely to become a standard offering in general dentistry, not just the domain of large labs or specialized digital centers.

### 7.4. Research Gaps and Future Studies

Despite the progress and enthusiasm, there remain significant research gaps and unanswered questions about 3D-printed splints. Addressing these through future studies will be crucial to fully establish printed splints as a gold-standard option. Key areas for future research include the following:

- Long-term clinical outcomes: While short-term and in vitro studies abound, we need more data on how 3D-printed splints perform over years of clinical use. How do printed splints hold up after 1 to 2 years of nightly wear? Do patients experience more frequent breakages or occlusal wear facets compared to traditional acrylic? Early indications (and analogies to printed dentures) suggest that annual or biannual replacement might be prudent, but robust longitudinal studies are lacking. Long-term follow-ups and post-market surveillance will help determine optimal recall intervals and whether printed splints can eventually match the proven longevity of well-made acrylic devices. Encouragingly, one review concluded that further optimization could allow printed splints to “match the longevity of milled splints” with continued material improvements [50]—a hypothesis that needs validation through long-term trials.
- Large-scale clinical trials: To date, most clinical evidence on printed splints comes from small cohort studies or case series. There is a need for larger randomized controlled trials comparing patient outcomes with 3D-printed splints versus conventional (or milled) splints in various scenarios—for instance, in managing TMD symptoms or sleep bruxism. Outcomes of interest include patient-reported comfort, adherence to use, reduction in symptoms (muscle pain, tooth wear, headache frequency), and any differences in side effects (e.g., tissue irritation). Such trials are now emerging. A notable example is an ongoing randomized trial comparing 3D-printed versus milled stabilization splints for TMD treatment (as published in a protocol format in the journal Trials). Results from these studies will provide high-level evidence to inform clinical guidelines. So far, smaller studies have found no significant differences in bruxism activity or TMD pain relief between printed and traditional splints over a few months, suggesting therapeutic equivalence [14]. Larger trials will confirm if this holds true broadly.
- Pediatric and orthodontic applications: Another gap lies in the use of 3D-printed splints for younger patients and unique applications. The pediatric trauma splint study by Wang et al. [126] demonstrated feasibility in children, but more research could explore 3D-printed appliances for mixed dentition, such as interim space maintainers or habit-breaking splints that accommodate growing arches. Orthodontic retention is another area—while clear thermoplastic retainers are common, one could envision custom rigid retainers or occlusal splints for post-orthodontic patients fabricated by printing. Do these printed retainers perform as well as vacuum-formed ones in maintaining tooth position? Questions like this remain to be studied. Additionally, the potential of printing splints that double as tooth movement guides (combining orthodontic and protective functions) could be a future research avenue.
- Standardization of testing and quality control: To compare materials and techniques, standardized testing protocols are needed. Researchers have noted a lack of uniform standards for evaluating printed splints’ mechanical properties and biocompatibility [133,134,135,136]. Future work could establish consensus methods (e.g., fatigue testing that simulates years of mastication, standardized fit evaluation metrics) so that new materials can be benchmarked reliably. Moreover, developing quality control tools—like AI-driven print error detection or built-in sensors that ensure a splint is fully cured—would enhance clinical safety and consistency. Research into printable RFID or QR codes on splints for tracking wear time or identifying material batches could also be envisioned to improve monitoring in clinical trials.
- Enhanced functionality: Finally, future splints may go beyond mechanical protection. Research might integrate smart sensors into splints to monitor bruxism in real-time or deliver therapeutic agents. Already, some prototypes exist of splints with embedded electronics that measure occlusal forces or jaw movements during sleep. Additive manufacturing could potentially incorporate micro-sensors or conductive elements inside the splint during printing. Although experimental, such intelligent splints could record patient compliance or the intensity of grinding, providing valuable data for clinicians and enabling biofeedback therapy. This cross-disciplinary direction, bridging material science, electronics, and AI, represents a cutting-edge frontier for dental devices.
- In summary, the future of 3D-printed splints in dentistry is bright, with rapid advancements on multiple fronts. Smart, adaptive materials and AI-driven processes promise to make splints more effective and easier to produce. At the same time, diligent research is underway to answer remaining questions about their long-term performance and to ensure they meet the highest standards of patient care. The ongoing convergence of digital technology and dental research will undoubtedly elevate the capabilities of occlusal splints in the coming years, benefiting practitioners and patients alike.

## 8. Conclusions

Three-dimensionally printed splints have emerged as a transformative innovation in dentistry, offering a compelling alternative to conventional acrylic appliances across prosthodontics, orthodontics, oral surgery, and beyond. This comprehensive review has examined their benefits—from unparalleled customization and streamlined production to digital reproducibility—as well as their current limitations in terms of material properties and processing requirements. The evidence to date indicates that, when fabricated and used appropriately, 3D-printed splints can achieve clinical outcomes on par with traditional splints while providing notable improvements in workflow efficiency and patient experience. In general practice, clinicians are leveraging these devices to protect teeth from bruxism and manage temporomandibular disorders—devices are tailored precisely to the patient’s occlusion. Specialists, too, are adopting printed splints: oral surgeons for surgical guides and stabilization plates, pediatric dentists for gentle trauma splints, and orthodontists for precise retention appliances. In all disciplines, the ability to design and manufacture intraoral splints digitally has introduced a new level of precision and flexibility in patient care.

Crucially, this review also highlights that the success of 3D-printed splints hinges on understanding and applying the proper protocols. Clinicians must be mindful of the technique’s nuances—from ensuring a quality digital impression and design to controlling print orientation to thorough post-curing. With adherence to best practices, issues like poor fit, inadequate strength, or biocompatibility concerns can be effectively mitigated. Moreover, as research drives improvements in printable resin chemistry and printer hardware, many of the current limitations (such as brittleness or initial costs) are gradually being overcome. For instance, new resin formulations are already demonstrating enhanced durability, and automated design software is reducing labor input, making the technology more accessible and reliable than ever before. Clinicians should also verify that their selected resins and printers comply with local regulatory approvals (e.g., CE marking, FDA clearance) before intraoral use.

In conclusion, 3D-printed splints represent a significant advancement in dental appliance therapy, marrying the precision of digital technology with the practical demands of clinical dentistry. They exemplify the ongoing digital transformation of our field—a transformation that brings efficiency, personalization, and innovation to everyday treatments. Patients benefit from splints that fit better and can be delivered faster, while clinicians benefit from the ability to fabricate appliances with consistency and control. These devices are particularly suitable for patients with moderate bruxism, proper occlusal trajectories, and without extreme parafunctional forces, whereas those with severe wear or significant asymmetry may still be better served by milled PMMA alternatives. Looking ahead, continued interdisciplinary research and development will further elevate the quality of 3D-printed splints, whether through smarter materials that adapt and endure or integrated digital systems that simplify their creation. Improved standardization and interoperability among CAD/CAM platforms, printers, and materials will be key to broader adoption, streamlining data flow and making these workflows accessible to smaller practices. With a strong foundation of positive clinical experience and growing scientific validation, 3D-printed splints are poised to become a mainstay in dental practice. Their clinical value today is clear, and their future potential is even greater—offering dental professionals a powerful tool to improve patient outcomes and embrace the full possibilities of digital dentistry.

## Figures and Tables

**Table 1:** Summary of Post-Processing Steps for 3D-Printed Splints.

Step	Purpose	Key Considerations	References
IPA Cleaning	Removes uncured resin	Use two separate baths of isopropyl alcohol (IPA), preferably with ultrasonic agitation for thorough removal.	[28,55,58,60]
Light Post-Curing	Increases strength and biocompatibility	Curing under 405 nm UV light (often heated) for 10–30 min completes polymerization and reduces toxicity.	[55,58,61,62]
Nitrogen Atmosphere	Enhances surface polymerization	Oxygen inhibition can reduce surface cure; curing in a nitrogen atmosphere improves hardness and strength.	[58,62,63]
Support Removal	Prepares final geometry	Supports are clipped, and nubs are ground or polished; avoid over-polishing fit surfaces.	[64]
Polishing	Improves comfort and reduces biofilm	Pumice and polishing paste are used; smoother surfaces reduce bacterial adhesion.	[65,66]
Sterilization (if needed)	Ensures safety for surgical use	Autoclaving may cause minimal distortion; low-temperature sterilization is preferred if resin is sensitive.	[66,67]

Abbreviations: IPA—isopropyl alcohol; UV—ultraviolet.

**Table 2:** Comparative overview of conventional, milled, and 3D-printed splints [8,13,27,32,33,61,64,68-72].

Aspect	Conventional (Heat-Cured/Self-Cured PMMA)	Milled CAD/CAM (Subtractive)	3D-Printed (Additive SLA/DLP)
Workflow and turnaround	Physical impressions ➜ stone models ➜ manual acrylic processing; 2–7 days typical.	Digital scan + CAD ➜ milled from pre-polymerized PMMA puck; chairside or 24 h via lab.	Scan + CAD ➜ print (≈1–2 h) ➜ wash + UV cure; same-day delivery feasible.
Core material and strength	Heat-cured PMMA; flexural strength ≈ 90–110 MPa.	Industrially polymerized PMMA; > 100 MPa, highest wear resistance.	Methacrylate photopolymers; 50–80 MPa (new resins up to ~90 MPa).
Biocompatibility/post-processing	Residual monomer possible; well-cured devices generally safe.	Negligible residual monomer; highly biocompatible.	Safe after thorough IPA wash + UV cure; technique-sensitive.
Clinical evidence	Decades of success for bruxism/TMD splints.	Strongest and longest-lasting; often the “gold standard” for heavy bruxers.	Randomized trial shows printed splints are non-inferior to milled for bruxism/TMD management.
Key advantages	Low material cost; easy repair; no high-tech equipment.	Superior strength and fit; digital files allow exact remakes; minimal chairside adjustment.	Fast, scalable, low resin waste; complex geometries; easy duplicate printing.
Main limitations	Labor-intensive; impression discomfort; polymerization shrinkage.	High capital cost; material wastage from puck; limited to rigid PMMA.	Slightly lower long-term strength; strict post-cure needed; equipment and resin costs.
